# No Immunogenicity of IPS Cells in Syngeneic Host Studied by *In Vivo* Injection and 3D Scaffold Experiments

**DOI:** 10.1155/2013/378207

**Published:** 2013-04-18

**Authors:** Suganya Thanasegaran, Zhao Cheng, Sachiko Ito, Naomi Nishio, Ken-ichi Isobe

**Affiliations:** Department of Immunology, Nagoya University Graduate School of Medicine, 65 Tsurumai-cho, Showa-ku, Nagoya 466-8550, Japan

## Abstract

Induced Pluripotent Stem Cells (IPSCs) open the great possibility to employ patient's own tissue to the previously incurable diseases. However these cells can be used in cell therapy only if they are not rejected when transplanted back into the syngeneic host. We found that the injection of iPSCs derived from different ages of mice into syngeneic C57BL/6 mice produced teratoma and was not rejected. Then we cultured iPSCs and myeloid differentiated iPSCs in three-dimensional porous scaffold and transplanted to C57BL/6 mice and BALB/C mice. After transplantation, we could observe the cell density inside the scaffold increased rapidly in syngeneic mice compared to the allogeneic mice indicating the favorable conditions supporting the growth of iPSCs *in vivo*. Unlike the allogeneic counterpart, we could not observe few infiltrating T cells inside the scaffold of syngeneic mice. These results contribute to the optimistic view of iPSCs for regenerative medicine in near future.

## 1. Introduction

Induced Pluripotent Stem Cells (iPSCs), one of the greatest inventions of the 21st century are nothing but embryonic stem cell like cells generated from somatic cells by the introduction of defined transcription factors Oct3/4, Sox2, Klf4, and c-Myc [1, 2]. Since iPSCs possess the ability to differentiate into various kinds of cells, successful differentiation of iPSCs to various somatic cells pertaining to different germ layers like neurons, cardiovascular progenitor cells, hepatocytes, and so forth is being reported repeatedly in recent years [3–6]. Unlike ES cells the use of IPSCs does not evoke moral or ethical objections [2, 7]. Also, ES cell transplantation causes HLA barrier, and their transplantation needs immunosuppressive drugs, which cause several side effects. Hence iPSCs have a great potential in regenerative medicine to treat innumerable diseases and clinical conditions. However the survival of transplanted iPSCs *in vivo* is questioned in a recent paper by Zhao et al. stating that transplantation of iPSCs derived from Mouse Embryonic Fibroblasts by retroviral reprogramming evokes an acute immune response in syngeneic recipients [8]. To treat damaged tissues or dysfunctional organs in elderly patients, it is necessary to establish iPSCs from the patient's own tissue. We succeeded in establishing iPS clones (aged iPSCs) using bone marrow (BM) of 21-month-old EGFP-C57BL/6 (EGFP-C57BL/6) mice that had been cultured for four days in granulocyte macrophage-colony stimulating factor (GM-CSF) [9]. 

## 2. Materials and Methods

### 2.1. Mice

C57BL/6 mice and BALB/C were purchased from SLC Japan. All mice were maintained in the Animal Research Facility at the Nagoya University Graduate school of Medicine under specific pathogen-free conditions and used according to institutional guidelines.

### 2.2. Cell Culture

MEF-iPSCs and aged iPSCs were previously established [9] from MEFs of C57BL/6 mice and bone marrow dendritic cells derived from 21-month-old EGFP-C57BL/6 mice (C14-Y01-FM1310sb) carrying pCAG-EGFP (CAGpromoter-EGFP). 15-month-old iPSCs were also established from Bone Marrow dendritic cells of 15 month old C57BL/6 mice. SNL76/7 feeder cells were clonally derived from the STO cell line transfected with G418^R^ and an LIF expression construct [10].

All iPS cells were maintained by culturing in KO-DMEM (Gibco) supplemented with 1% L-glutamine, 1% nonessential amino acids, 100 U/mL penicillin, 100 *μ*g/mL streptomycin, 5.5 mM 2-mercaptoethanol, and 15% FBS (iPS medium) at 37°C with 5% CO_2_. 

Myeloid-differentiated MEF-iPS cells were previously generated from C57BL/6 mice [9] and cultured in RPMI 1640 medium (10%FBS), 300 g/L L-glutamine, 100 U/mL penicillin, 100 *μ*g/mL streptomycin, and 50 mM 2-mercaptoethanol (Sigma Aldrich) containing 0.3% GM-CSF supernatant. (from murine GM-CSF producing Chinese hamster ovary cells, a gift from Dr T.Sudo, Toray Silicon, Tokyo, Japan). This medium is referred to as complete RPMI medium hereafter.

### 2.3. 3D Scaffolds

 3D scaffolds (code: LG/HP-BL005) were purchased from GC Biolabs Funakoshi, Japan. GC scaffold is a biodegradable material for the paradigm of stem cell and tissue engineering. It is a PLGA (poly D,L-Lactic acid glycolic acid) based product with validated biocompatibility. MEF-iPSCs and Myeloid-differentiated MEF-iPSCs were seeded (1 × 10^5^) into the scaffolds and cultured in 96-well plates with similar culture conditions as mentioned above. The scaffolds were transplanted into the dorsal flanks of syngeneic or allogeneic mice for different time periods and surgically operated. 

### 2.4. Teratoma Formation Assay

iPSCs were collected and washed twice with PBS, and a total of 100 *μ*L of the cell suspension (1 × 10^7^) were injected subcutaneously into the dorsal flank of C57BL/6 mice. Three weeks after injection the teratoma was surgically dissected from the mice.

### 2.5. Histological Analysis and Immunostaining

 The samples were fixed with 4% formaldehyde. The sections were stained with hematoxylin for 2 min, washed in tap water for 5 min, then stained with eosin for 4 min, and washed in tap water for 5 min. After dehydration, the sections were mounted with mounting medium (Malinol), observed, and then photographed using a Keyence BZ-8000 microscope (Osaka) or Olympus FSX 100 microscope. Frozen tissue samples and scaffolds were sectioned at 5–10 *μ*m thickness with the cryostat. The cryosections were then fixed in acetone, and nonspecific binding sites were blocked with 0.2% bovine serum albumin and 1% goat serum in PBS. The sections were then incubated with rat anti-mouse CD4 or anti-CD8 monoclonal antibody (eBioscience) conjugated to FITC and PE, respectively. After incubation the specimen was counterstained with Dapi. We observed and took pictures by using Nikon A1Rsi confocal Microscope.

### 2.6. Electron Microscope

Teratoma samples were immediately immersed in 2.5% glutaraldehyde and prepared for electron microscope as described previously [11]. 

### 2.7. Statistical Analysis

Statistical analysis was expressed as mean ± standard deviation (SD). Student's *t*-test was used to determine statistical significance. Results were considered significant if the *P* value was <0.05.

## 3. Results

**Figure 1:**
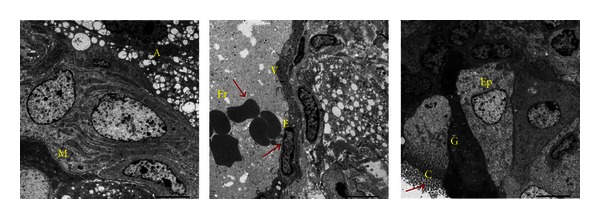
Three germ lines in clone 1 of MEF iPSCs. Electron microscopic observation of the teratoma showed different structures including muscle cell (M) and adipose cell (A), Venue (V) showing erythrocyte (Er) and endothelial cells (E) and ciliated columnar epithelial cell (Ep) and goblet cell (G) with cilia (C) (×1000).

In this study we analyzed the immunogenicity of iPSCs clones derived from different ages of mice. MEF iPSCs, aged-iPSCs derived from 21-month-old male were already described [9]. We added newly established iPSCs clones (clone 1 and clone 2) derived from 15 months old C57BL/6 mice, by selecting pluripotent markers (data not shown). All these iPSCs were established by retroviral reprogramming. We investigated the teratoma formation potential of different iPSCs clones by subcutaneous injection of the cells in C57BL/6 and BALB/c mice. We found that the injection of two clones of 21-month-old iPSCs and two clones of 15-month-old iPSCs resulted in teratoma after 3 weeks, whereas the teratoma forming ability in MEF iPSCs is 92.86%. All the teratoma continued to grow and did not regress. There was no teratoma formation by any of the iPSCs in allogeneic BALB/c mice (Table 1). We confirmed the presence of tissue types of all three germ layers by hematoxylin and Eosin staining in newly established 15-month-old iPSCs, clone 2 of 21-month-old iPSCs and clone 2 of MEF-iPSCs (data not shown). By performing Transmission Electron Microscopy (TEM), we confirmed the presence of different cell types pertaining to ectoderm, endoderm, and mesoderm in teratoma of MEF-derived iPSCs. The number of immune cells that could be observed by TEM is highly negligible (Figure 1).

**Figure 2:**
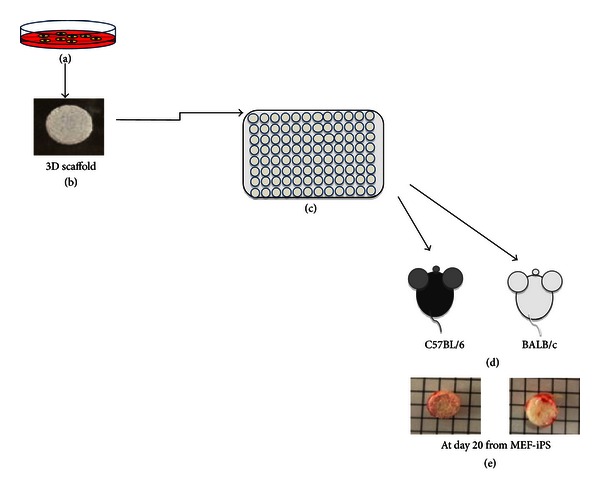
Overview of 3D culture in scaffold of iPSCs and transplantation to syngeneic and allogeneic mice for immune rejection analysis. Clone 1 of MEF iPSCs cultured on feeder (a) was trypsinyzed and washed and total of 1 × 10^5^cells of MEF iPSCs were seeded in the 3D scaffold (b). After 2 weeks of culture in 96-well plate (c), they were subcutaneously transplanted into C57BL/6 mice and BALB/c mice (d). After different time periods the scaffolds were removed from the mice and frozen in OCT (e).

**Figure 3:**
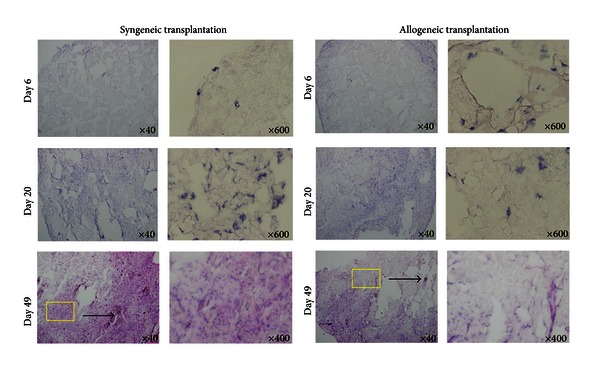
Growth of MEF iPSCs in scaffold transplanted in syngeneic mice and rejection of iPSCs in scaffold transplanted in allogeneic mice. MEF iPSCs (1 × 10^5^) were cultured in scaffolds for 2 weeks and transplanted into C57BL/6 mice or BALB/c mice. After different time periods the scaffolds were removed from the mice and frozen in OCT. Hematoxylin and Eosin staining of the scaffolds operated at day 6, 20, and 49 from the syngeneic and allogeneic host is shown.

**Figure 4:**
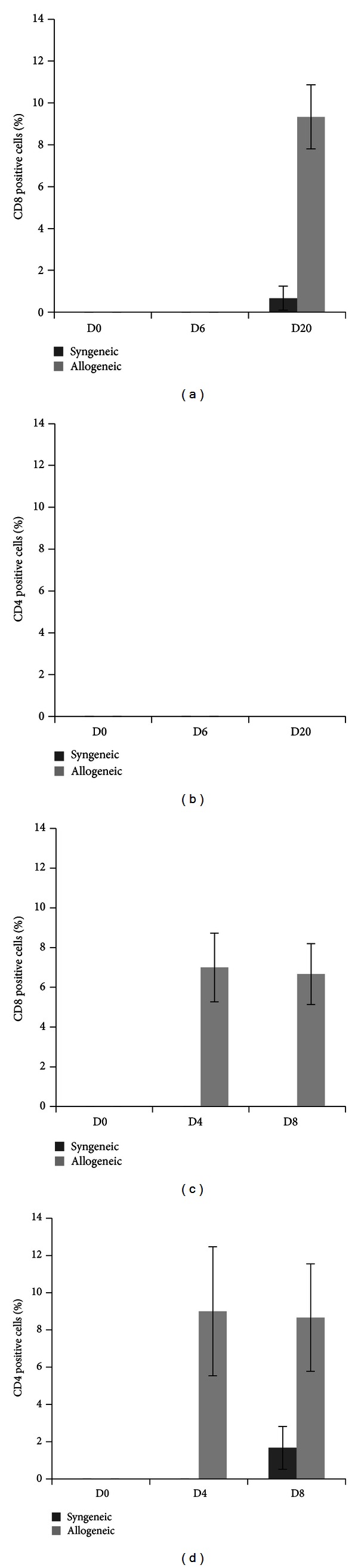
Lack of immunogenicity of MEF iPSCs in syngenic recipient. (a, b) MEF iPSCs (1 × 10^5^) derived from C57BL/6 were cultured in scaffolds for 2 weeks. They were transplanted into C57BL/6 or BALB/c mice. Tissue sections of the explanted scaffold were stained with FITC-labeled anti-CD4 and PE-labeled anti-CD8. The graph shows the average number of CD8 (a) or CD4 (b) positive cells at day 0, day 6, and day 20 (*n* = 3). (c, d) C57BL/6 derived splenocytes (2 × 10^6^) were injected into BALB/c or C57BL/6 mice as a primary challenge. After 2 weeks MEF-iPSCs, which were cultured inside scaffold for 2 weeks, were transplanted to immunized BALB/c or C57BL/6 mice. The graph shows the average number of CD8 or CD4 positive cells at day 0, day 6, and day 20 (*n* = 3). Error bars represent standard deviation.

Then, we try to evaluate immune cell infiltration into teratoma made by the transplantation of iPSCs either into C57BL/6 or BALB/c mice. The potential of teratoma formation can be evaluated only after 3 weeks in syngeneic host and failure of teratoma formation in allogeneic mice becomes difficult to comparative analysis of the teratoma tissue in syngeneic and allogeneic host backgrounds. We asked whether by any other means we could analyze the rate of acceptance of these cells inside the host even before a teratoma could be observed visually. Hence we made use of three-dimensional (3D) porous scaffold made by PLGA (poly D, L- Lactic acid glycolic acid) for the *in vitro *culture and transplantation of iPSCs subcutaneously into the host (Figure 2). We investigated the growth rate of iPSCs in syngeneic and allogeneic hosts. A total of 1 × 10^5^ cells of MEF iPSCs were seeded in the scaffold and after 2 weeks of continuous culture in 96-well plate; the scaffolds were washed in PBS and transplanted into syngeneic C57BL/6 and allogeneic BALB/c mice. At different days after transplantation, the scaffolds were removed (Figure 2(e)). Hematoxylin and Eosin staining of the scaffolds shows that the cell density inside the scaffolds transplanted to syngeneic C57BL/6 mice increases rapidly, whereas the scaffolds transplanted to allogeneic BALB/c mice had low cell density (Figure 3). We next investigated the T cell infiltration in transplanted scaffolds. Neither in BALB/c nor in C57BL/6 mice could we observe CD4 or CD8 T cell infiltration at day 6 after transplantation. However at day 20 there is increase in the number of CD8 T cells in allogeneic host compared to the syngeneic host (Figure 4(a)), but we could not observe a CD4 positive cell in the syngeneic and allogeneic host (Figure 4(b)). These results indicate that there is no induction of T cell infiltration by MEF iPSCs upon syngeneic transplantation. We also examined secondary challenge of MEF iPSCs in scaffold. We first injected a total of 2 × 10^6^ cells of splenocytes from C57BL/6 mouse into BALB/c or C57BL/6 mice as a primary challenge followed by transplantation of iPSCs cultured inside scaffold after 2 weeks. At day 4 and day 8, we could observe immediate increase in CD8 as well as CD4 T cell number in the allogeneic explants. In contrast, we could not detect any CD8 or CD4 positive T cells at day 4 and only a few CD4 cells could be detected at day 8 in syngeneic explants. These results signify that there is little or no evidence of immune response induced by iPSCs transplantation in syngeneic host even after secondary challenge (Figures 4(c) and 4(d)).

**Figure 5:**
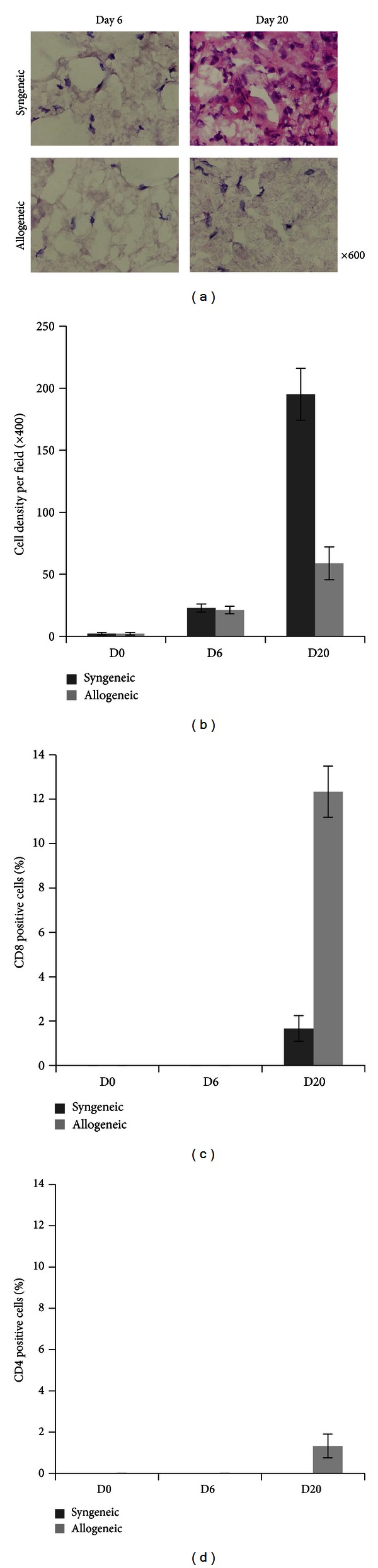
Lack of immunogenicity of myeloid differentiated MEF iPSCs in syngenic recipient. Myeloid differentiated MEF iPSCs (1 × 10^5^) cultured in scaffolds for 2 weeks. They were transplanted into C57BL/6 or BALB/c mice. (a) Hematoxylin and eosin staining of the scaffolds operated at day 6 and 20. (b) The number of cells in the scaffold was counted at a magnification of 400x. (*n* = 3). Tissue sections of the explanted scaffold are with FITC-labeled anti-CD4 and PE-labeled anti-CD8. The graph shows the average number of CD8 (c) or CD4 (d) positive cells at day 0, day 6 and day 20, respectively (*n* = 3). Error bars represent standard deviation.

In order to apply iPSCs from patient's own tissue, we must differentiate these iPSCs to diseased tissue cells and transplant these cells back to patient. Thus we next explored the immunogenicity of completely differentiated iPSCs. Hence, we made use of myeloid differentiated C57BL/6-derived MEF iPSCs, which were previously established [9]. Similar to the MEF-iPSCs scaffolds experiments, differentiated macrophages from MEF iPSCs were also cultured in 3D scaffolds and transplanted into syngeneic and allogeneic hosts for evaluating the immune response. We found that the cell proliferation rate was relatively slow and not significantly different in syngeneic and allogeneic hosts up to 6 days after transplantation. However after 20 days, the cell density in syngeneic explants was found to be 70% greater than the allogeneic one (Figures 5(a) and 5(b)). In the case of evaluating T cell infiltration, neither CD4 nor CD8 positive cells could be observed until 6 days after transplantation of the scaffolds in any of the recipients. But, after 20 days numerous number of infiltrating CD8 positive cells were detected in the scaffolds transplanted to BALB/c mouse, which accounts for 87% more than the syngeneic counterpart. However the number of CD4 positive cells that could be detected is highly negligible in both types (Figures 5(c) and 5(d)). These findings conclude that there is no significant immune response induced by myeloid differentiated MEF iPSCs when transplanted into syngeneic hosts.

## 4. Discussion

Zhao et al. have shown that iPSCs made by four retrovirus are immunogenic. They showed that teratomas developed by retrovirus-induced iPSCs were rejected by syngeneic mice by T cells [8]. They have shown that abnormal gene expression in some cells differentiated from iPSCs can induce T-cell-dependent immune response in syngeneic recipients. They implicated immunogenicity-causing Zg16 and Hormad1 genes [8]. Their results are contradictive to our results. Okita et al. commented on their results. Zhao et al. used only one line of iPSCs to compare immunogenicity [12]. One possibility of differences between our results and the results of Zhao et al. is that during the course of iPSCs production some iPSCs get copy number variation [13, 14], retro element stability and infrequent DNA rearrangement [15], and chromosomal aberrations [16] somatic mosaicism [17, 18]. These changes may induce neoantigens in iPSCs or after differentiation, which will be recognized by syngeneic host. These changes may be included in some cells in one clone, which are derived from fibroblasts in skin. In our experiments, only one in 14 trials of teratoma transplantation derived from MEF iPSCs was rejected, which might be recognized in syngeneic host by inducing T cells. However, all clones derived from bone marrow were not rejected by syngeneic host. We think that if there exist some iPSCs, which carry some abnormalities in genes and produced neoantigens, these cells are rejected by T cells in some stages of development. However, other iPSCs, which have normal genes, can grow in syngeneic host. Here we analyzed teratoma formation of several iPSCs clones derived from MEF or macrophages from different ages of mice. Almost all clones were accepted in syngeneic mice but not allogeneic mice. 

For clinical application, immunogenicity of differentiated cells must be examined. We [4, 19, 20] have reported model transplantation works of differentiated iPSCs into syngeneic mice and have shown that transplanted cells have been detected *in vivo*. Although we could detect differentiated iPSCs* in vivo*, the number of detected iPSCs was few. First possibility is that transplanted iPSCs might be low ability to grow *in vivo*, which might be dead *in vivo *and phagocytosed by macrophages. Second possibility is that transplanted iPSCs are diffused *in vivo*, thus it is difficult to detect by tissue staining. Third possibility is that differentiated iPSCs expressed altered-self, which might be rejected by immune T cells. Here we clearly showed that differentiated macrophages were not rejected by T cells of syngeneic mice by using scaffold transplantation. Thus our previous difficulties might be caused by migration of transplanted cells to other site, which induce the difficulty of detection of transplanted iPSCs. We could avoid diffusion of injected iPSCs *in vivo *by using scaffold. During the course of preparing manuscript, Araki et al. reported that terminally differentiated cells derived from iPSCs are not immunogenic. They used skin and bone marrow cells from chimera mice developed from iPSCs. Differentiated iPSCs-derived skin or bone marrow cells transplanted to syngeneic host were not rejected by T cells [21]. Considering these results and our results presented here, iPSCs have no immunogenicity in syngeneic recipient with or without differentiated form.

## 5. Conclusions

Here we showed that iPSCs are not rejected in syngeneic host. Nonimmunogenicity of iPSCs is not restricted to MEF-derived iPSCs. Aged-iPSCs are also not rejected in syngeneic mice. These findings favor the possibility to use iPSCs from patient own tissue to treat incurable diseases such as liver, lung, and kidney failure. 

## Figures and Tables

**Table 1:** 

Type of iPSC	Type of mouse	No. of mice	Teratoma after injection
21-month-aged iPSC clone I	BALB/c	4	0/4
MEF iPSC clone I	BALB/c	4	0/4
21-month-aged iPSC clone I	C57BL/6	22	22/22
21-month-aged iPSC clone II	C57BL/6	5	5/5
MEF iPSC clone I	C57BL/6	14	13/14
MEF iPSC clone II	C57BL/6	6	6/6
15-month-old iPSCs clone I	C57BL/6	12	12/12
15-month-old iPSCs clone II	C57BL/6	10	10/10
